# Dynamic impacts of COVID-19 on immune cells and inflammatory indicators in Chinese medical health workers: a retrospective longitudinal study

**DOI:** 10.3389/fcimb.2026.1782892

**Published:** 2026-04-20

**Authors:** Juanjuan Yang, Wei Jiang

**Affiliations:** 1 Department of Health Management, The Second Affiliated Hospital of Xi’an Jiaotong University, Xi’an, China; 2 Comprehensive Breast Care Center, The Second Affiliated Hospital of Xi’an Jiaotong University, Xi’an, China

**Keywords:** COVID-19, immune cells, inflammation indicators, medical health workers, psychological stress

## Abstract

**Background:**

The outbreak and ongoing impact of coronavirus disease 2019 (COVID-19) have significantly affected people’s physical and mental health, particularly among medical health workers. However, the effects of COVID-19 on the immunity of this population remain unclear.

**Methods:**

This retrospective longitudinal study analyzed data from blood routine examinations of medical health workers conducted in 2019, 2020, 2022, and 2024. Data were collected from 1,818 participants, with subgroup analyses stratified by gender.

**Results:**

White blood cell (WBC), lymphocyte (LYM), and eosinophil (EOS) counts increased significantly in the post-COVID-19 year compared with the pre-COVID-19 year. Neutrophil (NEUT) counts decreased in COVID-19 year 1 and COVID-19 year 3 but returned to pre-COVID-19 year levels in the post-COVID-19 year. Monocyte (MONO) counts and monocyte-to-lymphocyte ratio (MLR) decreased in COVID-19 year 1, increased to pre-COVID-19 year levels in COVID-19 year 3, and reached their highest levels in the post-COVID-19 year. Basophil (BASO) counts and hemoglobin-to-red blood cell distribution width ratio (HRR) were significantly higher in COVID-19 year 1, COVID-19 year 3, and post-COVID-19 year than in pre-COVID-19 year. Platelet-to-lymphocyte ratio (PLR) in pre-COVID-19 year was significantly higher than in COVID-19 year 1 and post-COVID-19 year, but not significantly different from COVID-19 year 3. Neutrophil-to-lymphocyte ratio (NLR) significantly decreased in post-COVID-19 year when compared with pre-COVID-19 year. Aggregate index of systemic inflammation (AISI) in pre-COVID-19 year was significantly higher than in COVID-19 year 1 and COVID-19 year 3, but significantly lower than in post-COVID-19 year. Systemic inflammation response index (SIRI) decreased in COVID-19 year 1, but up to their pre-COVID-19 year levels in COVID-19 year 3 and post-COVID-19 year. Stratified analysis showed that the impacts of COVID-19 on immune cells and inflammation indicators were more pronounced in female.

**Conclusion:**

Immune cells and inflammatory markers in medical health workers underwent changes between 2019 and 2024, particularly among female. Increased attention should be paid to the immune function and mental health of medical health workers.

## 1 Introduction

The outbreak of novel coronavirus disease 2019 (COVID-19) has posed a catastrophic threat to public health in China and even the world (Silk et al., 2023). Meanwhile, the outbreak and ongoing situation of COVID-19 have exerted a significant influence on people’s physical and mental health, particularly on medical health workers (Pappa et al., 2020; Mostafa et al., 2021). During the COVID-19 pandemic, the sharp increase in workload and the heightened risk of COVID-19 infection have exerted tremendous psychological stress on medical health workers (Shi et al., 2020). As reported in a previous research, medical health workers, especially women, nurses, and frontline medical health workers, suffered from stress-related mental disorders, such as more than 50% of participants reported symptoms of depression, more than 50% reported distress and 44.6% reported anxiety (Lai et al., 2020). Another research reported that 53% of the emergency medical technicians perceived a moderate stress level (Amro et al., 2022). It can be seen that medical health workers suffered from tremendous mental and psychological stress during the COVID-19 pandemic.

Psychological stress has a significant impact on the quantity, distribution, and function of immune cells (Jiang et al., 2017; Yang and Jiang, 2020). Psychological stress regulates immunity through stress responses, which primarily involve the secretion of norepinephrine, epinephrine, and glucocorticoids by activating the sympathetic nervous system (SNS) and the hypothalamic-pituitary-adrenocortical axis (HPA) (Jiang et al., 2017; Obeagu, 2025). In a general way, chronic stress has a negative effect on the immune system (Jia et al., 2025).

Blood routine examination is a basic blood test used to assess an individual’s physical health and immune status. White blood cells (WBC) serve as a non-specific indicator of inflammation (Shafiee et al., 2017). Among WBC, neutrophils (NEUT) are mainly responsible for fighting bacterial infections, lymphocytes (LYM) are mainly responsible for defending against viral infections and humoral immunity, monocytes (MONO) are mainly responsible for clearing dead cells and fighting certain bacterial infections, and elevated eosinophils (EOS) are associated with allergies and parasitic infections, while elevated basophils (BASO) may be associated with allergies, chronic inflammation, or hematological diseases. Some new inflammatory indicators are derived from the blood routine examination, such as platelet-to-lymphocyte ratio (PLR), neutrophil-to-lymphocyte ratio (NLR), monocyte-to-lymphocyte ratio (MLR), aggregate index of systemic inflammation (AISI), systemic inflammation response index (SIRI), hemoglobin-to-red blood cell distribution width ratio (HRR) (Liu et al., 2024; Xi et al., 2024). Our previous studies showed that the post-stress frontline medical workers of COVID-19 had lower MONO count, NLR, MLR, and NEUT percentage than the controls or the pre-stress, however, but those inflammation indicators returned to normal after 10 months of frontline work for COVID-19 (Yang et al., 2021; Yang et al., 2022). A previous study indicated that BASO counts decreased during COVID-19 year 1, LYM decreased and MLR increased in COVID-19 year 2 of airport workers (Cao et al., 2024). However, the impacts of COVID-19 on immune cells and inflammation indicators among medical health workers in China remain unknown.

Here, we aimed to study the impacts of COVID-19 on immune cells and inflammation indicators among medical health workers in China. We selected four timepoints to capture distinct phases of the pandemic: 2019 as the pre pandemic baseline (pre-COVID-19 year); 2020 as the early pandemic period (acute stress and initial disruption, COVID-19 year 1); 2022 as the late pandemic period (prolonged stress, vaccination rollout, and repeated outbreaks, COVID-19 year 3); and 2024 as the post pandemic era (after the lifting of major public health restrictions, post-COVID-19 year). This temporal stratification allows us to evaluate short term disruption, potential recovery and long-term immune adaptation rather than simply comparing pre and post pandemic periods.

## 2 Materials and methods

### 2.1 Participants

This retrospective longitudinal study included medical health workers (frontline physicians, nurses, technicians) from the Second Affiliated Hospital of Xi’an Jiaotong University during Jan 2019 and Dec 2024. They underwent checkups at the Second Affiliated Hospital of Xi’an Jiaotong University. Participants were required to undergo COVID-19 SARS-CoV-2 virus nucleic acid test before their checkups, and only those with negative test results were allowed to proceed with the checkups in 2020 and 2022. Participants who were pregnant or breastfeeding, malignant tumors, acute infections (fever, cough, sore throat, etc.), immune-related diseases, and taking immunosuppressants were excluded from this study. This study was approved by the Ethics Committee of Xi’an Jiaotong University College of Medicine in accordance with the Declaration of Helsinki.

### 2.2 Demographic and clinical data collection

The demographic and clinical data were collected from the hospital information system of our hospital, including gender, age, hypertension history, diabetes history, body mass index (BMI), and smoking history. Blood routine examination was performed using Sysmex XN-9000 Auto Hematology Analyzer (Sysmex Corporation, Kobe, Japan). WBC count, NEUT count, LYM count, MONO count, EOS count, BASO count, platelet count, hemoglobin (Hb), and red blood cell distribution width (RDW) were collected.

### 2.3 Calculation of inflammation indicators

The PLR is equal to the platelet count divided by the LYM count. The NLR is equal to the NEUT count divided by the LYM count. The MLR is equal to the MONO count divided by the LYM count. The SIRI is calculated by multiplying NEUT count by MONO count and then dividing by the LYM count. The AISI is calculated by multiplying NEUT count, platelet count, and MONO count, and then dividing by the LYM count. The HRR is equal to the Hb divided by the RDW.

### 2.4 Statistical analysis

Data were analyzed using IBM SPSS Statistics for Windows (version 24.0; IBM Corp., Armonk, NY, USA). Non-normally distributed continuous variables were presented as median and interquartile range (IQR), and categorical variables were presented as absolute values and percentages. Kolmogorov-Smirnov test was used for normality test. The Friedman Non-parametric Repeated Measures ANOVA test was used to compare immune cells and inflammatory indicators among different years. If the differences are statistically significant, *post-hoc* pairwise comparisons were performed using Dunn’s test, with Bonferroni correction applied to adjust for multiple comparisons. For the overall Friedman test, Kendall’s W (coefficient of concordance) was reported. This fixed categorization was maintained across all follow up years to ensure consistency in subgroup comparisons. Statistical significance was set at *P* < 0.05.

## 3 Results

### 3.1 Demographic and clinical characteristics of participants

**Figure 1:**
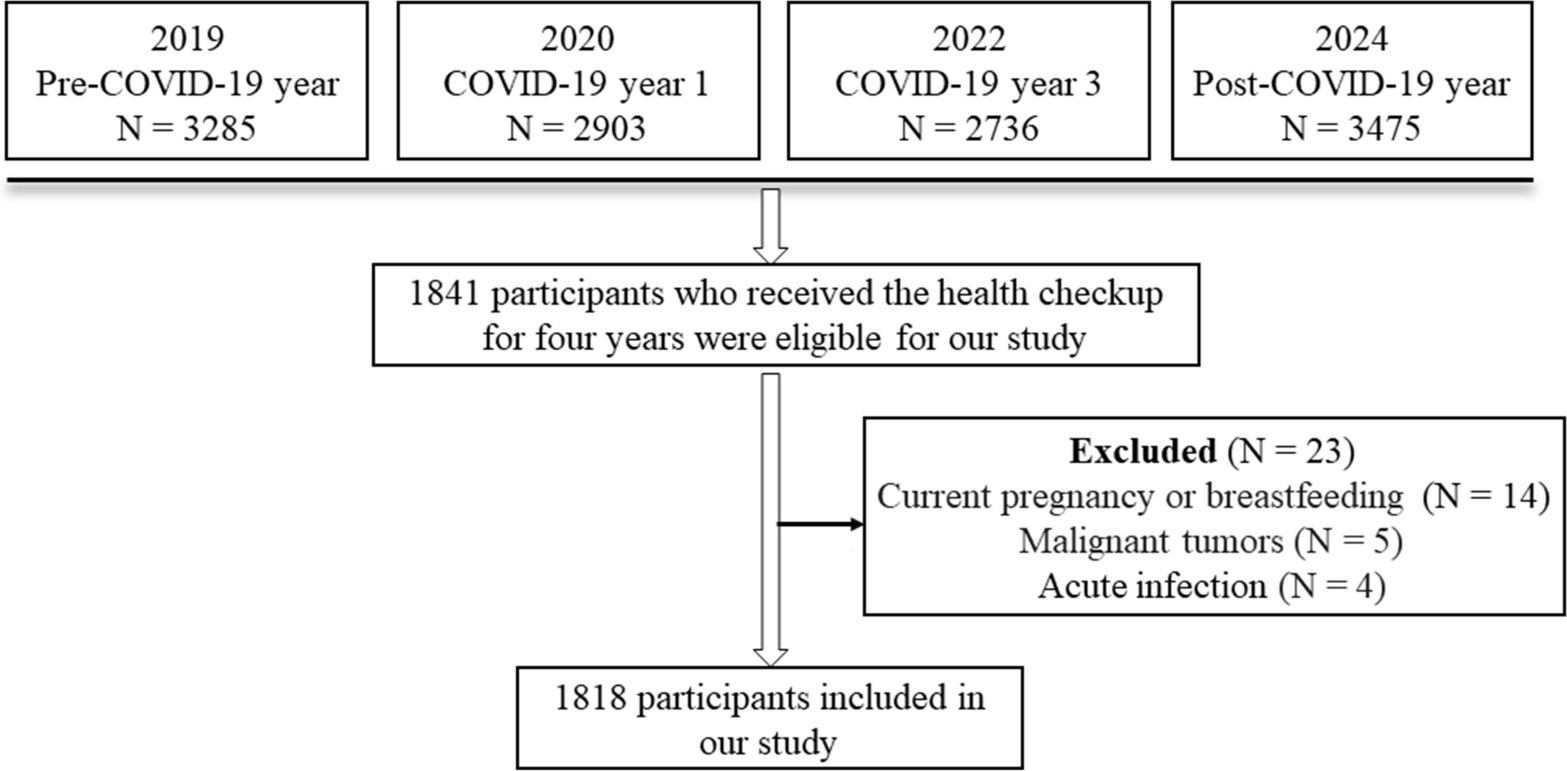
Flowchart.

In general, 3285 participants underwent health examinations in 2019, 2903 participants underwent health examinations in 2020, 2736 participants underwent health examinations in 2022, and 3475 participants underwent health examinations in 2024 (**Figure 1**). A total of 1841 participants who received the health checkup in four years were eligible for our study (**Figure 1**). After screening, 14 participants were excluded for current pregnancy or breastfeeding, 5 participants were excluded for malignant tumors, and 4 participants were excluded for acute infection (**Figure 1**). Finally, 1818 participants were included in our study. The demographic and clinical characteristics of 1818 participants were presented in **Table 1**.

**Table 1:** Demographic and clinical characteristics of participants.

Variable	Total 1818 participants
Age, years	43.00 (33.00, 57.00)
Age < 60, n (%)	1433 (78.82%)
Age ≥ 60, n (%)	385 (21.18%)
Male, n (%)	439 (24.15%)
Female, n (%)	1379 (75.85%)
Hypertension, n (%)	181 (9.96%)
Diabetes, n (%)	46 (2.53%)
Current smoker, n (%)	21 (1.16%)
Former smoker	8 (0.44%)
Never smoker	1789 (98.40%)
BMI, kg/m^2^	23.30 (21.30, 25.40)
BMI < 18.5, n (%)	53 (2.92%)
18.5 ≤ BMI < 24.0, n (%)	1022 (56.22%)
BMI ≥ 24.0, n (%)	743 (40.87%)

BMI, body mass index.

### 3.2 The impacts of COVID-19 on immune cells and inflammation indicators among medical health workers

**Figure 2:**
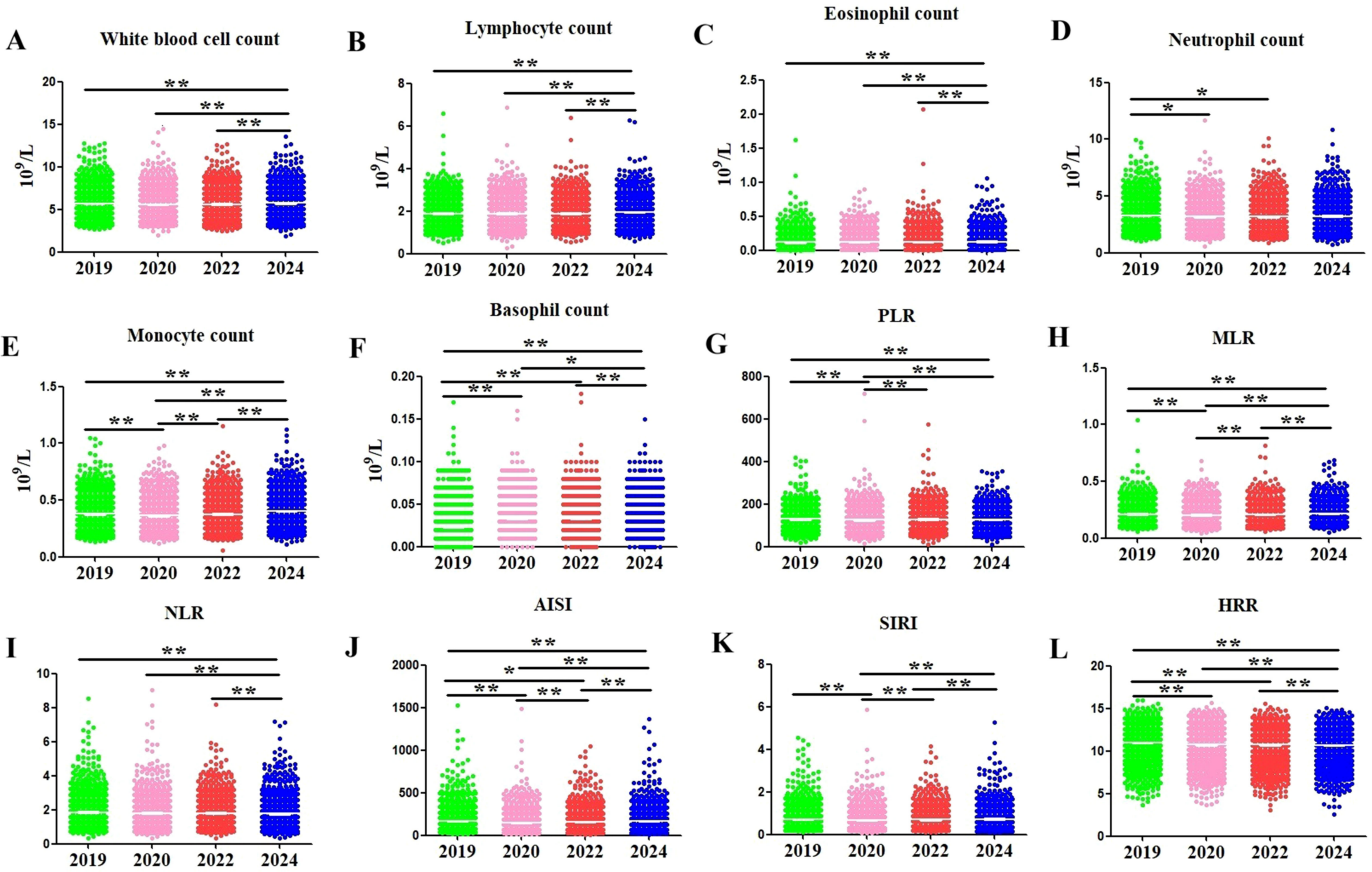
Dynamic changes of immune cells and inflammation indicators from 2019 to 2024. **(A)** White blood cells count; **(B)** Lymphocytes count; **(C)** Eosinophils count; **(D)** Neutrophils count; **(E)** Monocytes count; **(F)** Basophils count; **(G)** Platelet-to-lymphocyte ratio (PLR); **(H)** Monocyte-to-lymphocyte ratio (MLR); **(I)** Neutrophil-to-lymphocyte ratio (NLR); **(J)** Aggregate index of systemic inflammation (AISI); **(K)** Systemic inflammation response index (SIRI); **(L)** Hemoglobin-to-red blood cell distribution width ratio (HRR). *P < 0.05, **P < 0.01.

There were no significant differences in WBC count, LYM count, and EOS count among pre-COVID-19 year, COVID-19 year 1, and COVID-19 year 3, but they significantly increased in the post-COVID-19 year (adjusted *P* = 0.002, <0.001, <0.001, respectively, **Table 2**; **Figures 2A–C**). NEUT count decreased in COVID-19 year 1 and COVID-19 year 3, but reached their pre-COVID-19 year levels in the post-COVID-19 year (adjusted *P* = 0.026, 0.045, 1.000, respectively, **Table 2**, **Figure 2D**). MONO count decreased in COVID-19 year 1, but increased and reached pre-COVID-19 year levels in COVID-19 year 3, and reached their highest levels in the post-COVID-19 year (adjusted *P* = <0.001, 1.000, <0.001, respectively, **Table 2**, **Figure 2E**). The BASO count was significantly higher in COVID-19 year 1, COVID-19 year 3, and post-COVID-19 year than in pre-COVID-19 year (adjusted all *P* < 0.001, **Table 2**, **Figure 2F**). As for inflammation indicators, the values of PLR in pre-COVID-19 year were significantly higher than in COVID-19 year 1 and post-COVID-19 year (adjusted all *P* < 0.001, **Table 2**, **Figure 2G**), but not significantly different from COVID-19 year 3 (adjusted *P* = 0.497, **Table 2**, **Figure 2G**). MLR in pre-COVID-19 year was significantly higher than in COVID-19 year 1 (adjusted *P* < 0.001, **Table 2**, **Figure 2H**), not significantly different from COVID-19 year 3 (adjusted *P* = 1.000, **Table 2**, **Figure 2H**), but significantly lower than in the post-COVID-19 year (adjusted *P* < 0.001, **Table 2**, **Figure 2H**). There were no significant differences in the values of NLR among pre-COVID-19 year, COVID-19 year 1, and COVID-19 year 3, but significantly decreased in the post-COVID-19 year (adjusted *P* = 0.050, 0.163, <0.001, respectively, **Table 2**, **Figure 2I**). The values of AISI in pre-COVID-19 year were significantly higher than in COVID-19 year 1 and COVID-19 year 3 (adjusted *P* < 0.001 and 0.018, respectively, **Table 2**, **Figure 2J**), but significantly lower than in the post-COVID-19 year (adjusted *P* = 0.004, **Table 2**, **Figure 2J**). The values of SIRI decreased in COVID-19 year 1, but up to their pre-COVID-19 year levels in COVID-19 year 3 and post-COVID-19 year (adjusted *P* = <0.001, 0.730, 0.112, respectively, **Table 2**, **Figure 2K**). The values of HRR were significantly higher in COVID-19 year 1, COVID-19 year 3, and post-COVID-19 year than in pre-COVID-19 year (adjusted all *P* < 0.001, **Table 2**, **Figure 2L**).

**Table 2:** Dynamic changes of immune cells and inflammation indicators from 2019 to 2024.

Variable	Gender	2019 Pre-COVID-19 year	2020 COVID-19 year 1	2022 COVID-19 year 3	2024 Post-COVID-19 year	Kendall’s W	*P*
WBC count (10^9^/L)	Total	5.45 (4.63, 6.45)	5.40 (4.55, 6.35)	5.42 (4.57, 6.38)	5.60 (4.73, 6.58) **^##$$^	0.008	<0.001
	Male	6.03 (5.19, 6.98)	5.85 (4.91, 6.78)	6.01 (5.19, 6.89)	6.24 (5.33, 7.16) ^#^	0.007	0.035
	Female	5.30 (4.50, 6.25)	5.29 (4.47, 6.19)	5.23 (4.42, 6.19)	5.36 (4.59, 6.35) *^##$$^	0.007	<0.001
LYM count (10^9^/L)	Total	1.82 (1.51, 2.16)	1.81 (1.49, 2.18)	1.80 (1.50, 2.17)	1.89 (1.55, 2.26) **^##$$^	0.015	<0.001
	Male	1.91 (1.62, 2.26)	1.93 (1.61, 2.29)	1.94 (1.62, 2.30)	2.03 (1.70, 2.41) ^#^	0.008	0.019
	Female	1.78 (1.48, 2.13)	1.78 (1.48, 2.13)	1.75 (1.48, 2.12)	1.84 (1.52, 2.21) **^##$$^	0.014	<0.001
EOS count (10^9^/L)	Total	0.08 (0.05, 0.14)	0.09 (0.05, 0.14)	0.09 (0.05, 0.15)	0.10 (0.06, 0.15) **^##$$^	0.009	<0.001
	Male	0.11 (0.07, 0.19)	0.12 (0.07, 0.19)	0.12 (0.08, 0.20)	0.12 (0.08, 0.21)	0.002	0.456
	Female	0.08 (0.05, 0.13)	0.08 (0.05, 0.13)	0.08 (0.05, 0.13)	0.09 (0.06, 0.14) **^##$$^	0.009	<0.001
NEUT count (10^9^/L)	Total	3.08 (2.48, 3.82)	3.00 (2.43, 3.77) *	3.03 (2.43, 3.74) *	3.08 (2.46, 3.80)	0.002	0.008
	Male	3.36 (2.77, 4.23)	3.30 (2.61, 3.99)	3.37 (2.78, 4.11)	3.40 (2.77, 4.20)	0.003	0.331
	Female	2.99 (2.41, 3.68)	2.92 (2.38, 3.66)	2.89 (2.35, 3.61) *	2.99 (2.40, 3.70)	0.003	0.015
MONO count (10^9^/L)	Total	0.36 (0.30, 0.44)	0.35 (0.29, 0.42) **	0.36 (0.29, 0.43) ^##^	0.38 (0.31, 0.48) **^##$$^	0.048	<0.001
	Male	0.42 (0.34, 0.49)	0.39 (0.33, 0.46)	0.41 (0.35, 0.50)	0.45 (0.36, 0.54) **^##$$^	0.032	<0.001
	Female	0.35 (0.29, 0.42)	0.34 (0.28, 0.41) **	0.35 (0.29, 0.41) ^##^	0.37 (0.30, 0.45) **^##$$^	0.042	<0.001
BASO count (10^9^/L)	Total	0.02 (0.00, 0.04)	0.03 (0.02, 0.04) **	0.03 (0.02, 0.04) **	0.03 (0.02, 0.04) **^#$$^	0.075	<0.001
	Male	0.03 (0.00, 0.04)	0.03 (0.02, 0.05) **	0.03 (0.02, 0.05) **	0.04 (0.02, 0.05) **	0.053	<0.001
	Female	0.02 (0.00, 0.04)	0.03 (0.02, 0.04) **	0.03 (0.02, 0.04) **	0.03 (0.02, 0.04) **^$$^	0.075	<0.001
Hb (g/L)	Total	138.00 (130.00, 148.00)	136.00 (128.00, 146.00) **	135.00 (127.00, 145.00) ** ^##^	135.00 (127.00, 145.00) ** ^##^	0.060	<0.001
RDW (%)	Total	12.50 (12.10, 13.10)	12.70 (12.20, 13.20) **	12.50 (12.10, 13.10) ^##^	12.60 (12.20, 13.20) **^$$^	0.020	<0.001
Platelet (10^9^/L)	Total	225.00 (190.00, 262.00)	218.00 (181.00, 255.00) **	223.00 (187.00, 259.00) ^##^	228.00 (192.00, 266.00) *^##$$^	0.020	<0.001
PLR	Total	123.47 (100.00, 151.46)	118.65 (95.23, 146.04) **	122.66 (99.12, 150.48) ^##^	121.07 (97.67, 148.82) **^##^	0.009	<0.001
	Male	110.58 (88.44, 134.69)	106.67 (84.57, 131.13)	108.51 (86.15, 133.53)	105.00 (85.61, 133.90)	0.004	0.146
	Female	127.13 (104.76, 157.23)	123.04 (100.93, 151.01) **	126.28 (103.90, 156.25) ^##^	125.00 (102.01, 153.55) **^$$^	0.009	<0.001
MLR	Total	0.20 (0.16, 0.24)	0.19 (0.16, 0.24) **	0.20 (0.16, 0.24) ^##^	0.21 (0.17, 0.25) **^##$$^	0.017	<0.001
	Male	0.21 (0.18, 0.26)	0.21 (0.17, 0.25)	0.22 (0.17, 0.26)	0.22 (0.18, 0.27) ^##^	0.010	0.004
	Female	0.20 (0.16, 0.24)	0.19 (0.15, 0.23) **	0.19 (0.16, 0.23) ^##^	0.20 (0.17, 0.25) **^##$$^	0.017	<0.001
NLR	Total	1.69 (1.32, 2.16)	1.64 (1.31, 2.11)	1.65 (1.31, 2.14)	1.63 (1.27, 2.07) **^##$$^	0.007	<0.001
	Male	1.73 (1.36, 2.23)	1.68 (1.36, 2.15)	1.74 (1.36, 2.23)	1.68 (1.26, 2.15)	0.004	0.168
	Female	1.68 (1.31, 2.14)	1.64 (1.30, 2.10)	1.63 (1.30, 2.10)	1.61 (1.27, 2.05) **^##$$^	0.007	<0.001
AISI	Total	131.65 (94.40, 199.52)	123.15 (84.78, 183.58) **	129.95 (90.50, 191.82) *^##^	141.88 (97.82, 203.73) **^##$$^	0.025	<0.001
	Male	144.52 (104.52, 218.30)	136.34 (90.60, 193.06)	147.02 (102.06, 211.12)	158.44 (103.64, 231.08) ^##^	0.010	0.005
	Female	127.64 (90.51, 192.48)	119.34 (82.49, 178.80) **	125.04 (85.99, 183.91) *^##^	137.64 (96.16, 193.12) ^##$$^	0.020	<0.001
SIRI	Total	0.60 (0.44, 0.85)	0.57 (0.42, 0.81) **	0.59 (0.43, 0.84) ^##^	0.62 (0.44, 0.87) ^##$$^	0.010	<0.001
	Male	0.69 (0.52, 0.99)	0.65 (0.49, 0.94)	0.71 (0.53, 1.02)	0.74 (0.53, 1.04) ^#^	0.006	0.040
	Female	0.57 (0.41, 0.81)	0.54 (0.39, 0.77) **	0.56 (0.41, 0.77)	0.58 (0.43, 0.81) ^##$$^	0.007	<0.001
HRR	Total	11.01 (10.15, 12.02)	10.83 (9.92, 11.76) **	10.85 (9.92, 11.79) **	10.76 (9.85, 11.73) **^##$$^	0.045	<0.001
	Male	12.58 (11.80, 13.52)	12.33 (11.49, 13.05) **	12.36 (11.53, 13.01) **	12.20 (11.40, 12.98) **	0.025	<0.001
	Female	10.71 (9.85, 11.43)	10.50 (9.65, 11.26) **	10.53 (9.62, 11.25) **	10.48 (9.62, 11.18) **	0.035	<0.001

WBC, white blood cell; NEUT, neutrophil; LYM, lymphocyte; MONO, monocyte; EOS, eosinophil; BASO, basophil; Hb, Hemoglobin; RDW, red blood cell distribution width; PLR, platelet-to-lymphocyte ratio; NLR, neutrophil-to-lymphocyte ratio; MLR, monocyte-to-lymphocyte ratio; AISI, aggregate index of systemic inflammation (neutrophil count × platelet count × monocyte count/lymphocyte count); SIRI, systemic inflammation response index (neutrophil count × monocyte count/lymphocyte count); HRR, hemoglobin-to-red blood cell distribution width ratio. *P < 0.05 vs pre-COVID-19 year, **P < 0.01 vs pre-COVID-19 year,^#^*P* < 0.01 vs COVID-19 year 1, ^##^*P* < 0.01 vs COVID-19 year 1, ^$^*P* < 0.01 vs COVID-19 year 3, ^$$^*P* < 0.01 vs COVID-19 year 3.

### 3.3 Subgroup analyses

Subgroup analyses were performed by gender (male and female). Subgroup analysis revealed that the changes in WBC count, LYM count, EOS count, MONO count, BASO count, PLR value, MLR value, NLR value, SIRI value, and HRR value in the female subgroup were consistent with the main effect (**Table 2**). NEUT count decreased significantly in COVID-19 year 3 when compared with pre-COVID-19 year in the female subgroup (**Table 2**). The values of AISI in pre-COVID-19 year were significantly higher than in COVID-19 year 1 and COVID-19 year 3 in the female subgroup (**Table 2**). The changes in BASO count and HRR value in the male subgroup were consistent with the main effect (**Table 2**). There were no significant differences in EOS count, NEUT count, NLR value, and PLR value among pre-COVID-19 year, COVID-19 year 1, COVID-19 year 3, and in post-COVID-19 year in the male subgroup (**Table 2**). The WBC count, LYM count, MLR value, SIRI value, and AISI value in post-COVID-19 year were significantly higher than in COVID-19 year 1 in the male subgroup (**Table 2**). The MONO count in post-COVID-19 year was significantly higher than in pre-COVID-19 year, COVID-19 year 1, and COVID-19 year 3 in the male subgroup (**Table 2**).

## 4 Discussion

This is the first study to investigate the dynamic impacts of COVID-19 on immune cells and inflammation indicators among medical health workers. Our study found that the WBC count, NEUT count, LYM count, MONO count, EOS count, BASO count, PLR, NLR, MLR, SIRI, AISI, and HRR of medical health workers underwent changes from 2019 to 2024, especially for female.

During the COVID-19 pandemic, medical health workers underwent the sharp increase in workload, the high risk of COVID-19 infection, the lack of time for exercise, the isolation from family, as well as the excessive demands and expectations from society and hospitals, all of the above led to mental and psychological stress (Shi et al., 2020; Magnavita et al., 2021; Thapa and Pradhan, 2024). Medical health workers had a higher proportion of stress related psychiatric symptoms than nonmedical health workers during the COVID-19 pandemic, such as anxiety, depression, and insomnia (Zhang et al., 2020). A meta-analysis showed that during the pandemic, the proportion of anxiety among medical health workers was 23.2%, while the proportion of depression was 22.8% (Pappa et al., 2020). Psychological stress affects the immune system through stress response, including HPA and SNS (Huang et al., 2025). Our previous study found that participants with psychiatric symptoms had higher WBC and NEUT counts than controls (Yang et al., 2023). Psychotic disorder was also associated with inflammatory markers, such as IL-6 (Mongan et al., 2023). Animal experiments have also confirmed that restraint stress alters the number and function of immune cells in mice through the HPA and SNS (Jiang et al., 2017; Yang et al., 2024). He et al. found that frontline medical workers for treating patients of COVID-19 had significantly lower absolute numbers and percentages of peripheral blood CD19^+^ B cells (He et al., 2023). For the dynamic impacts of COVID-19 on peripheral blood immune cells, Cao et al. found the LYM count decreased while the MLR increased in COVID-19 year 2 when compared with pre- COVID-19 among airport workers (Cao et al., 2024). Our study found that the WBC count, NEUT count, LYM count, MONO count, EOS count, BASO count, PLR, NLR, MLR, SIRI, AISI, and HRR of medical health workers underwent changes from 2019 to 2024. Therefore, more attention should be paid to the immune function and mental health of medical health workers.

After the outbreak of the COVID-19 pandemic, China implemented a policy of universal vaccination against COVID-19. However, vaccination affects the body’s immune cells, especially LYM (Foster et al., 2023; Zhao et al., 2023). As we mentioned earlier, medical health workers lacked physical exercise during the COVID-19 pandemic, and physical exercise had a significant influence on immune function (Fiuza-Luces et al., 2024). During the COVID-19 pandemic or after the Chinese government announced the lifting of prevention and control measures for Class A infectious diseases caused by COVID-19, most Chinese medical health workers had been infected with COVID-19. Clinical evidences suggest that the immune system also underwent some changes after COVID-19 infection (Ranjbar et al., 2024; Sidiropoulou et al., 2025). Information on COVID 19 vaccination status was not available in this retrospective dataset. Given that vaccination can influence immune parameters, this represents a limitation of the study. Future prospective studies should incorporate the factor of vaccination history in order to better delineate its contribution to the observed immune changes. We also lacked detailed information about prior SARS‑CoV‑2 infection history, including the time and severity, which may influence longitudinal immune profiles. This precludes us from distinguishing the effects of the infection from other factors related to the pandemic.

Our stratified analysis showed the impacts of COVID-19 on immune cells and inflammation indicators were more sensitive among female. Similarly, He et al. found that the changes in absolute numbers and percentages of peripheral blood CD19^+^ B cells of frontline medical workers for treating patients of COVID-19 were more significant in female than in male, which was consistent with our results (He et al., 2023). Psychosocial stress has a greater impact on the immunity of female, which is attributed to the different stress responses between genders (Canto-de-Souza et al., 2025). On one hand, psychological stress can activate the HPA axis to release glucocorticoids, and then inhibit the secretion of gonadotropin-releasing hormone, thereby reducing the level of estradiol (Chrousos, 2010). Through negative feedback regulation, estradiol further stimulates the hypothalamus, leading to the release of corticotropin-releasing hormone and ultimately resulting in a further increase in glucocorticoids (Chrousos, 2010). However, testosterone has a relatively small effect on the HPA axis (Chrousos, 2010; Bekhbat and Neigh, 2018). On the other hand, the more severe mental stress burden experienced by female medical health workers may be one of the reasons for the more pronounced immune changes in women during the COVID-19 pandemic (Lai et al., 2020).

Our research has some limitations. Firstly, our research lacks data on psychiatric symptoms, immune cell subsets, and cytokines of participants. Secondly, although we collected baseline information on BMI, hypertension, diabetes, and smoking status, these factors were not included as covariates in the main analysis due to the use of a non-parametric repeated measures design. Future studies that employ mixed-effects models or adjust for time-varying confounders may provide additional insight into the independent effects of stress related to COVID-19 on immune parameters. Thirdly, the absence of a non-healthcare worker control group limits our ability to distinguish the effects of occupational stress from general pandemic related stressors. Future studies comparing healthcare workers with non-healthcare populations are warranted to isolate occupation specific effects. Finally, as this is a retrospective study, it cannot establish a causal relationship.

## 5 Conclusion

Our study found that the WBC count, NEUT count, LYM count, MONO count, EOS count, BASO count, PLR, NLR, MLR, SIRI, AISI, and HRR of medical health workers underwent changes from 2019 to 2024, particularly for female. More attentions should be paid to the immune function and mental health of medical health workers.

## Data Availability

The original contributions presented in the study are included in the article/supplementary material. Further inquiries can be directed to the corresponding author.
